# Supplementary material for: Optimization of the Vacuum Microwave Assisted Extraction of the Natural Polyphenols and Flavonoids from the Raw Solid Waste of the Pomegranate Juice Producing Industry at Industrial Scale
Source: Molecules. 2021 Feb 16;26(4):1033. doi: 10.3390/molecules26041033 (PMC7919679; doi:10.3390/molecules26041033)
Supplement: Supplementary file 1 [file molecules-26-01033-s001.pdf]

(a)

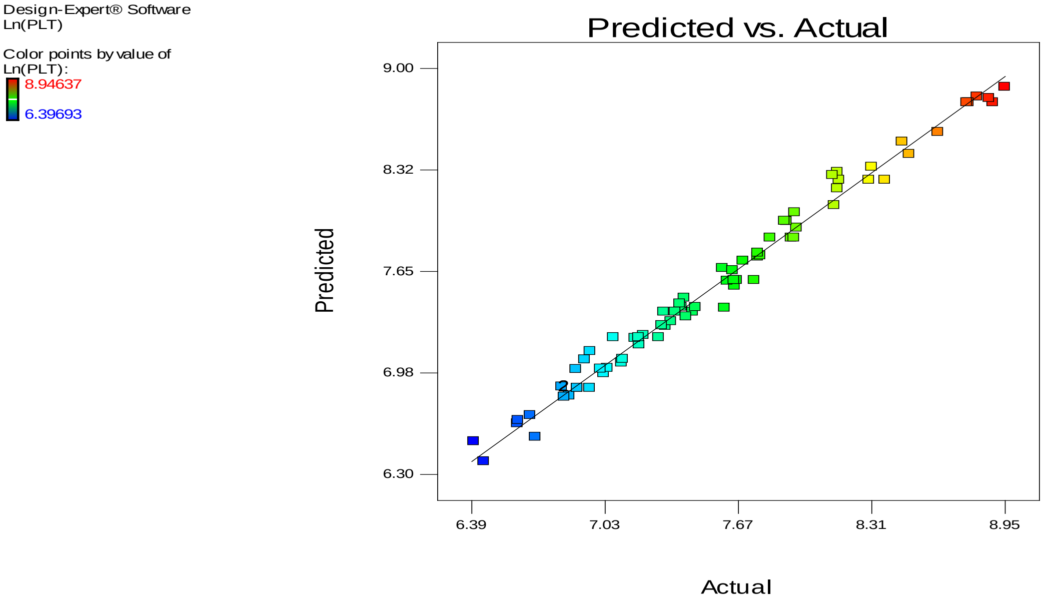

(b)

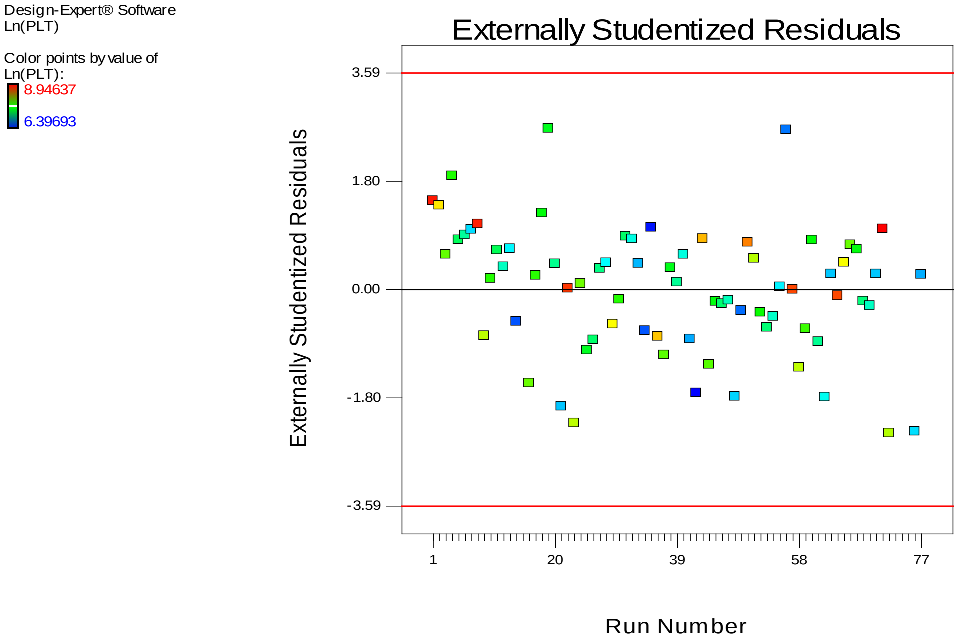

Figure S1. Predicted vs Actual values plot and Externally Studentized Residuals plots of natural logarithm of PP TPE/t response

(i)

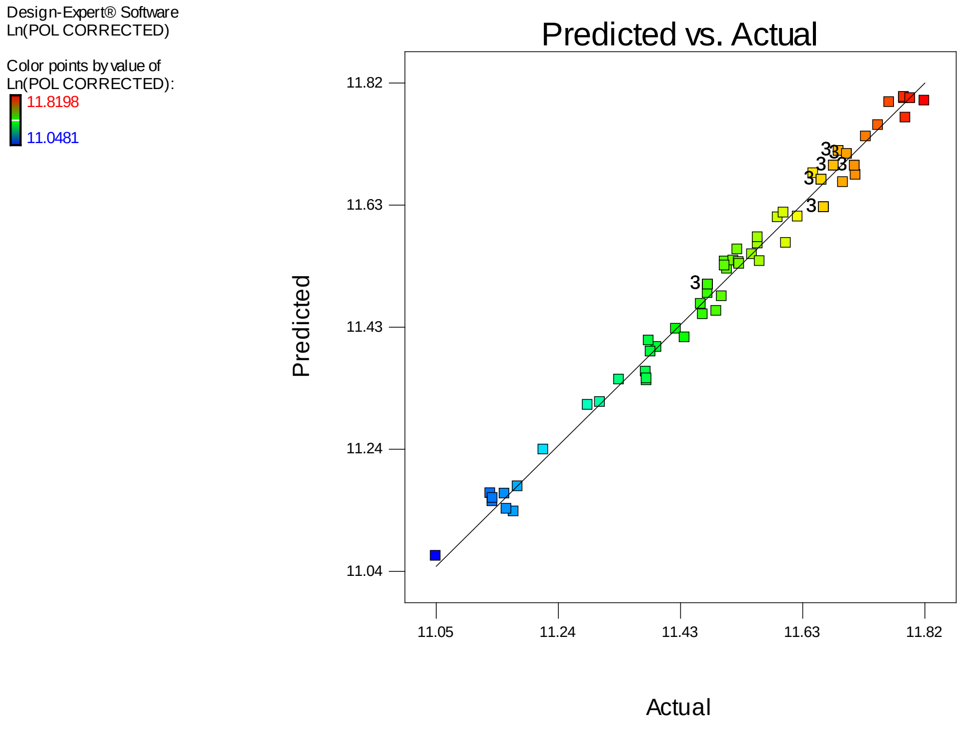
Design-Expert® Software
Ln(POL CORRECTED)
Color points by value of
Ln(POL CORRECTED):
11.8198
11.0481
Predicted vs. Actual
Predicted
11.82
11.63
11.43
11.24
11.04
Actual
11.05
11.24
11.43
11.63
11.82

(ii)

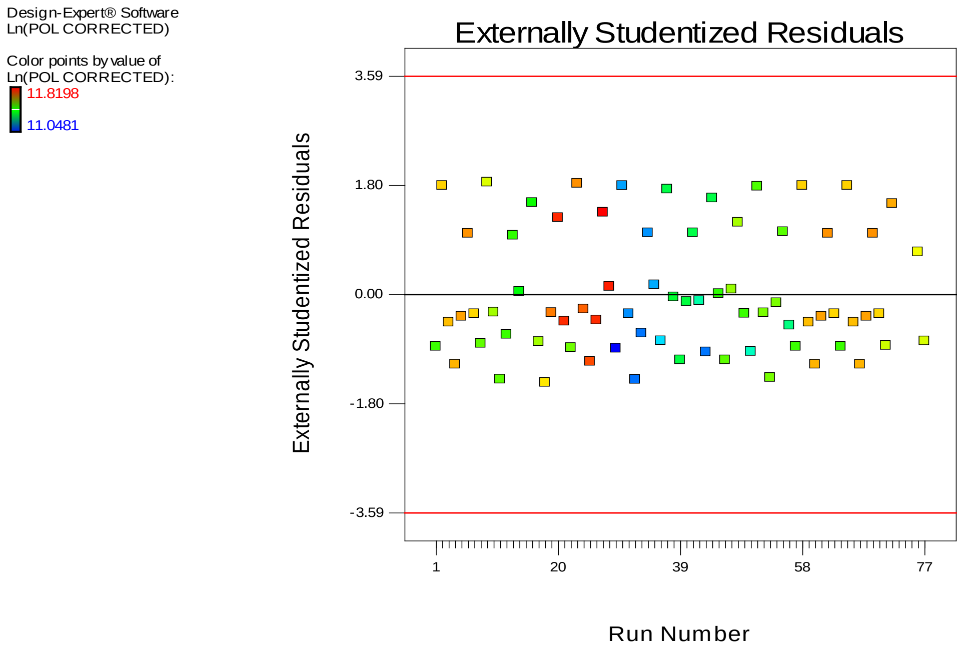
Design-Expert® Software
Ln(POL CORRECTED)
Color points by value of
Ln(POL CORRECTED):
11.8198
11.0481
Externally Studentized Residuals
Externally Studentized Residuals
3.59
1.80
0.00
-1.80
-3.59
Run Number
1
20
39
58
77

(iii)

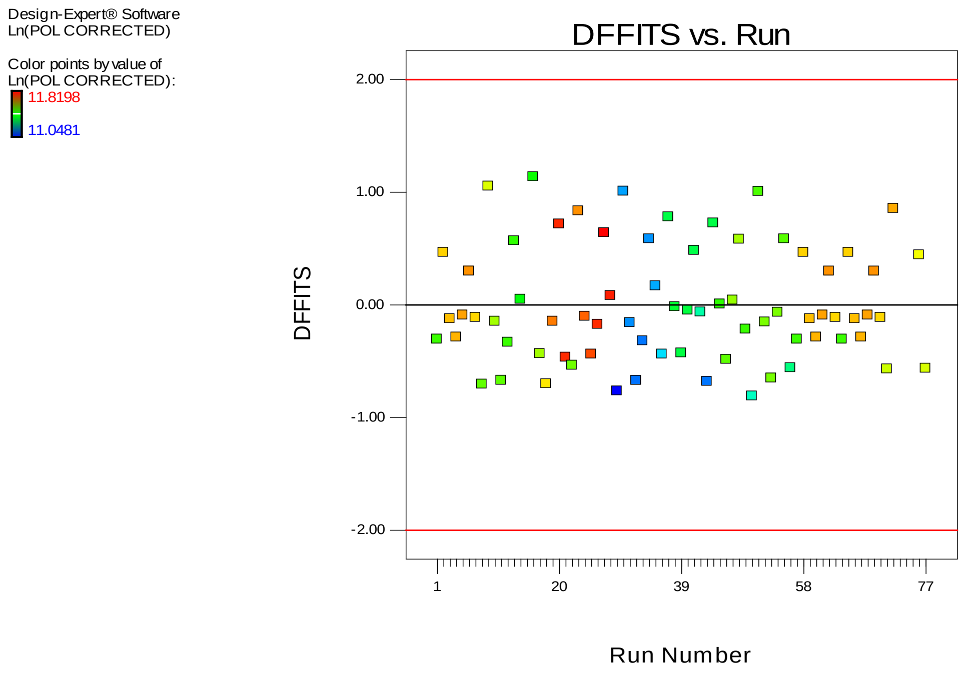

(iv)

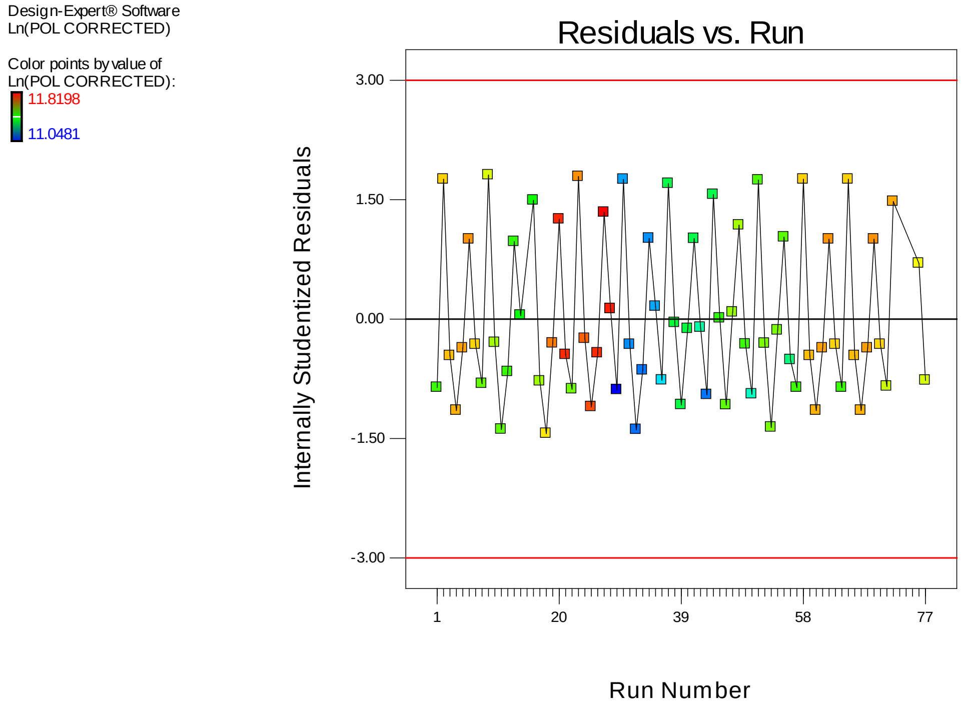

Figure S2. i) Predicted vs Actual values plot ii) Externally Studentized Residuals plot iii) DFFITS vs Run and iv) Residuals vs. Run plots of natural logarithm PP TPE response

(i)

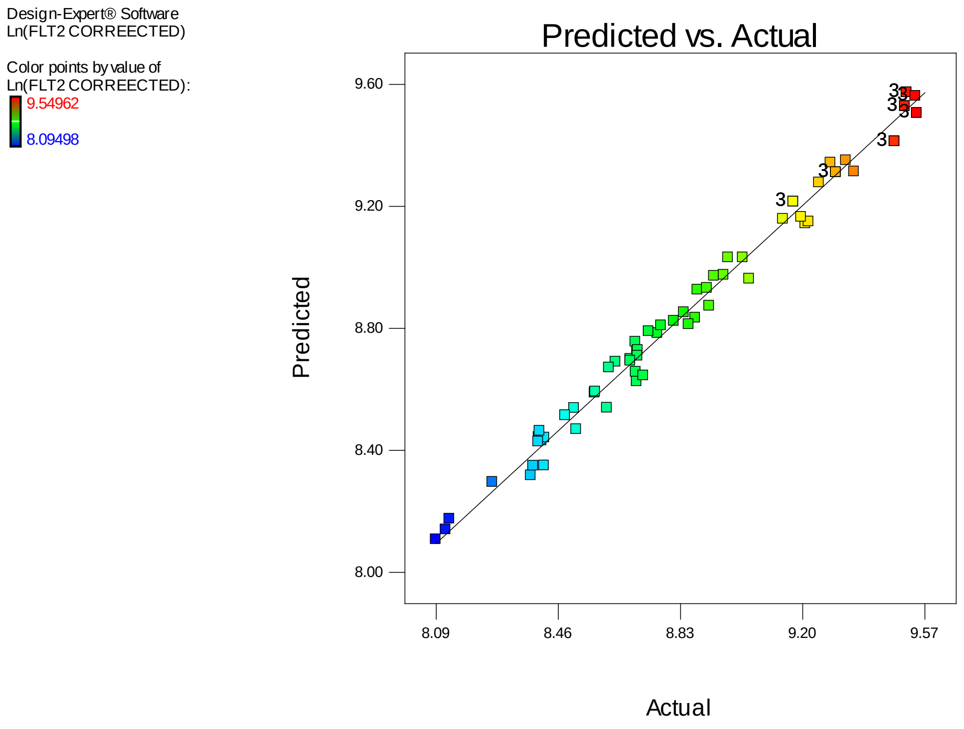
Design-Expert® Software
Ln(FLT2 CORREECTED)
Color points by value of
Ln(FLT2 CORREECTED):
9.54962
8.09498
Predicted vs. Actual
Predicted
Actual
9.60
9.20
8.80
8.40
8.00
8.09
8.46
8.83
9.20
9.57

(ii)

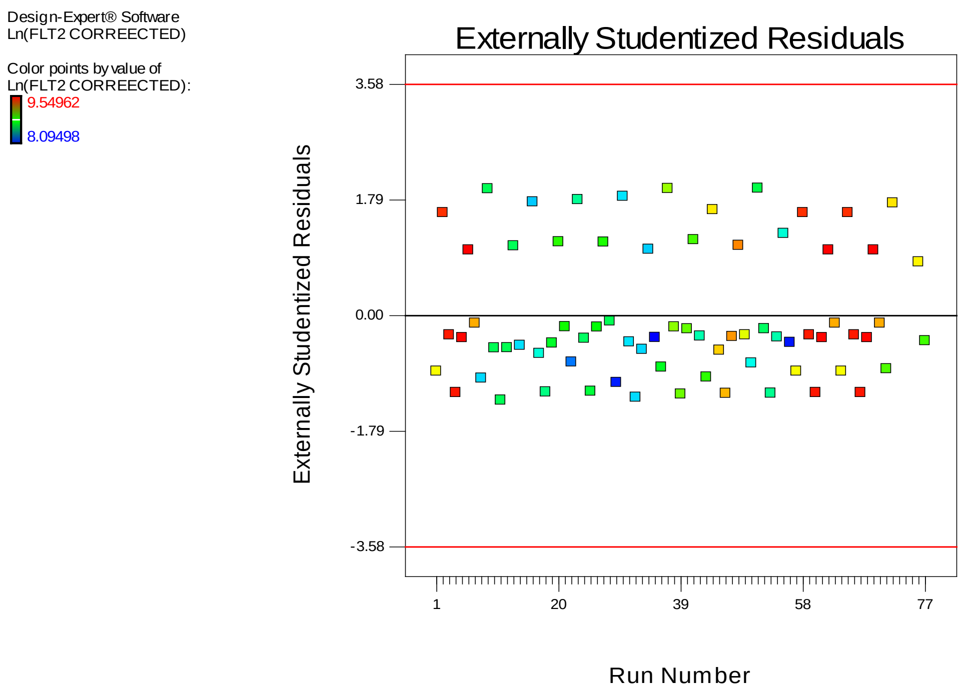
Design-Expert® Software
Ln(FLT2 CORREECTED)
Color points by value of
Ln(FLT2 CORREECTED):
9.54962
8.09498
Externally Studentized Residuals
Externally Studentized Residuals
Run Number
3.58
1.79
0.00
-1.79
-3.58
1
20
39
58
77

(iii)

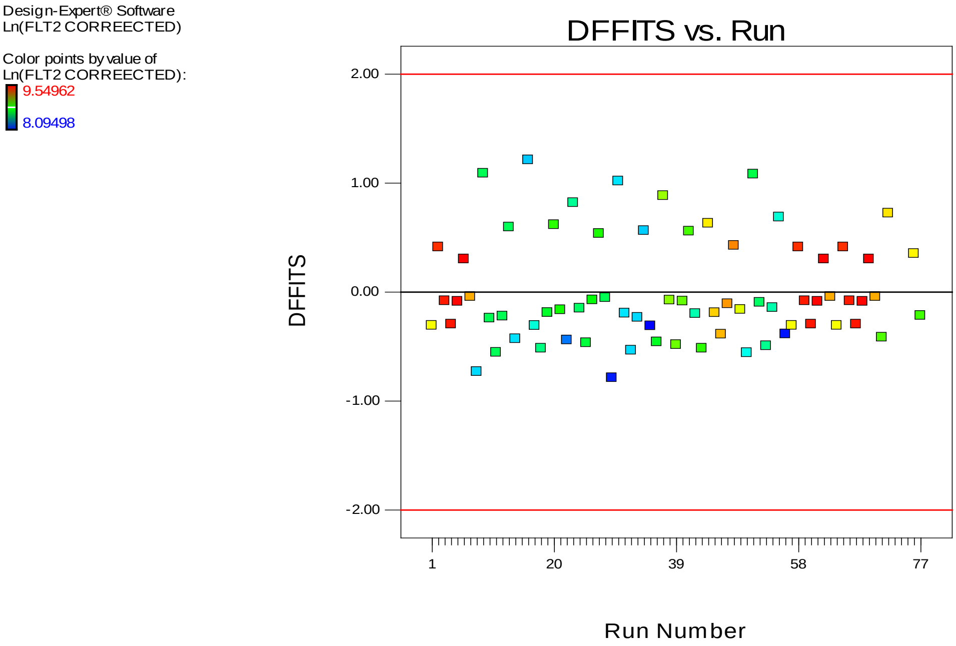

(iv)

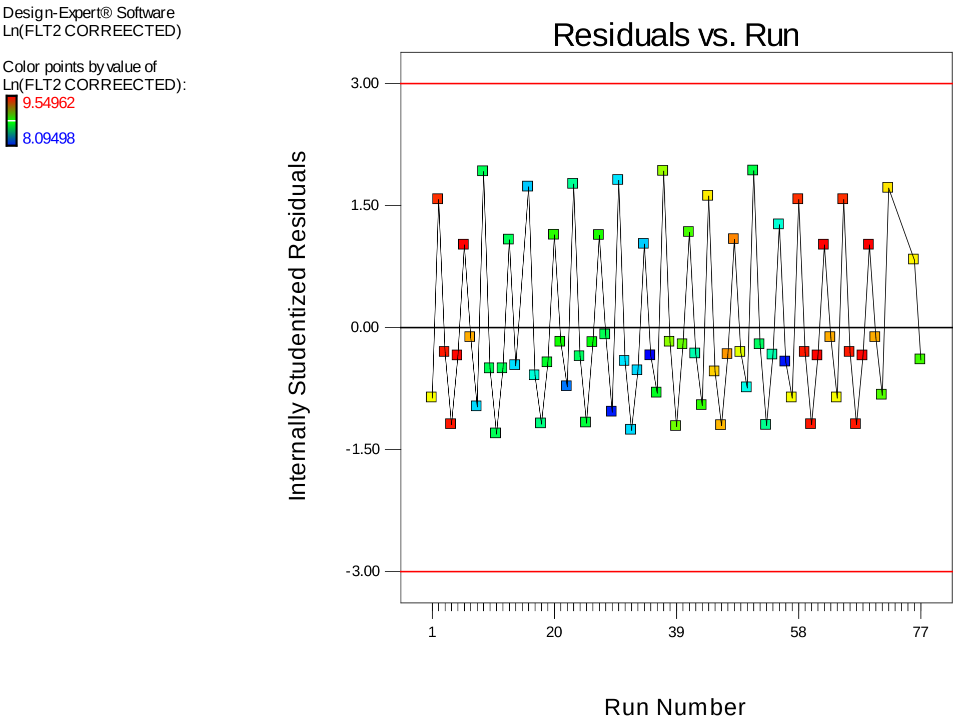

Figure S3. **i)**Predicted vs Actual values plot ii)Externally Studentized Residuals plot (iii) DFFITS vs Run and (iv) Residuals vs. Run plots for the natural logarithm of PP TFE response

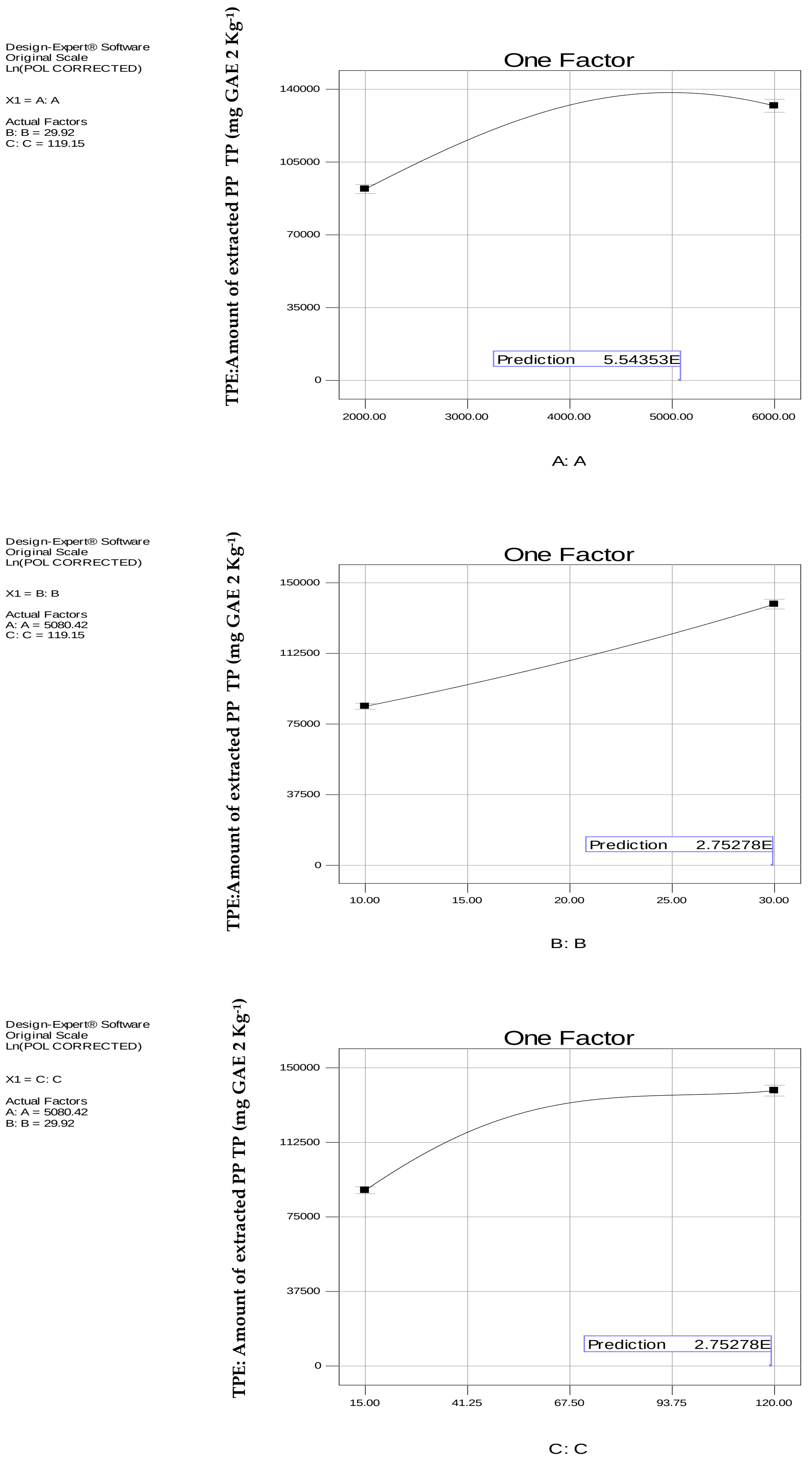

Figure S4. The effect of the individual extraction parameters A, B, C on the extracted amount of total polyphenols TPE.

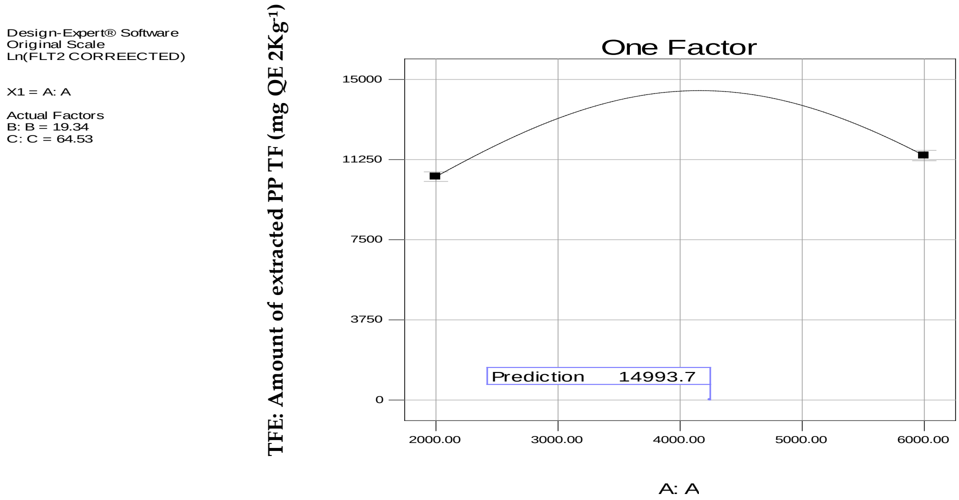

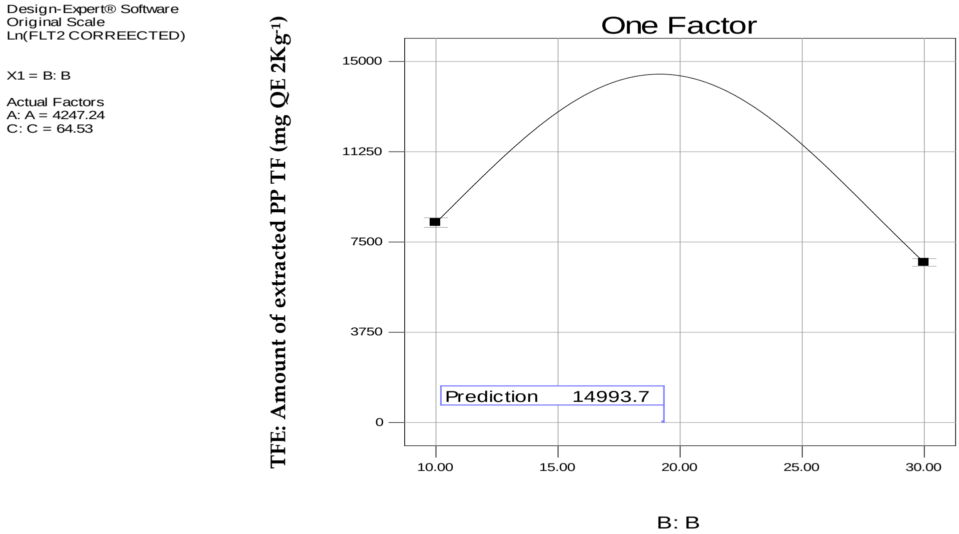

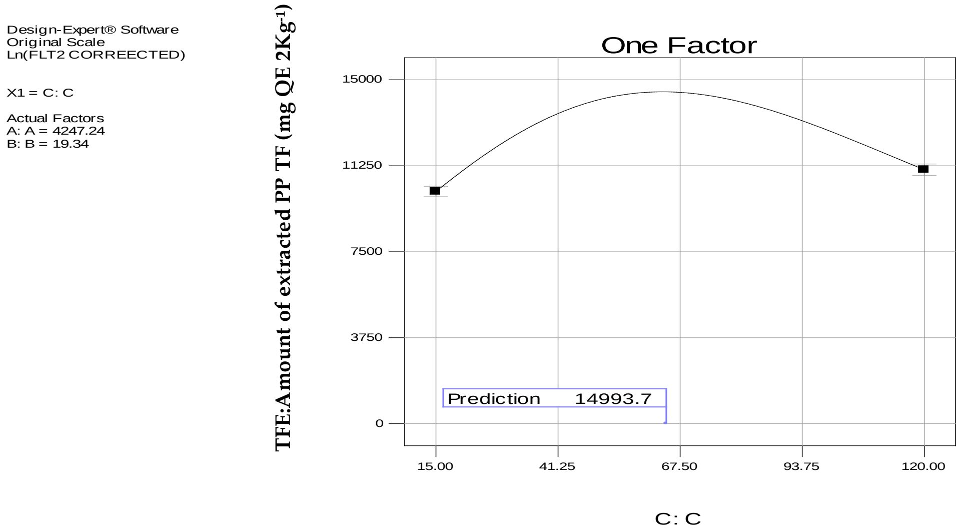

Figure S5. The effect of the individual extraction parameters A, B, C on the extracted amount of TFE

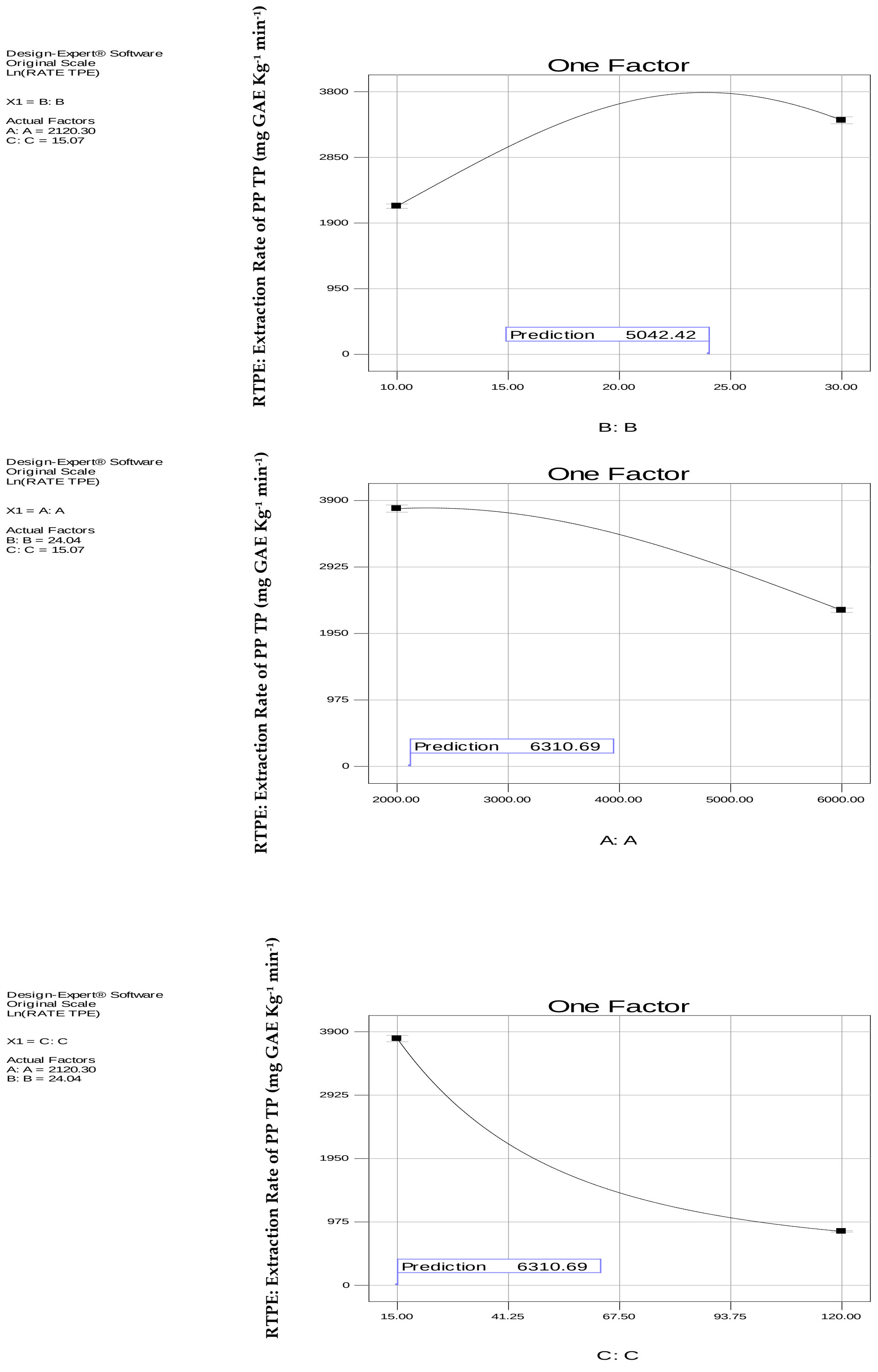

Figure S6. The effect of the individual extraction parameters A, B, C on the extraction rate of PP total polyphenols.

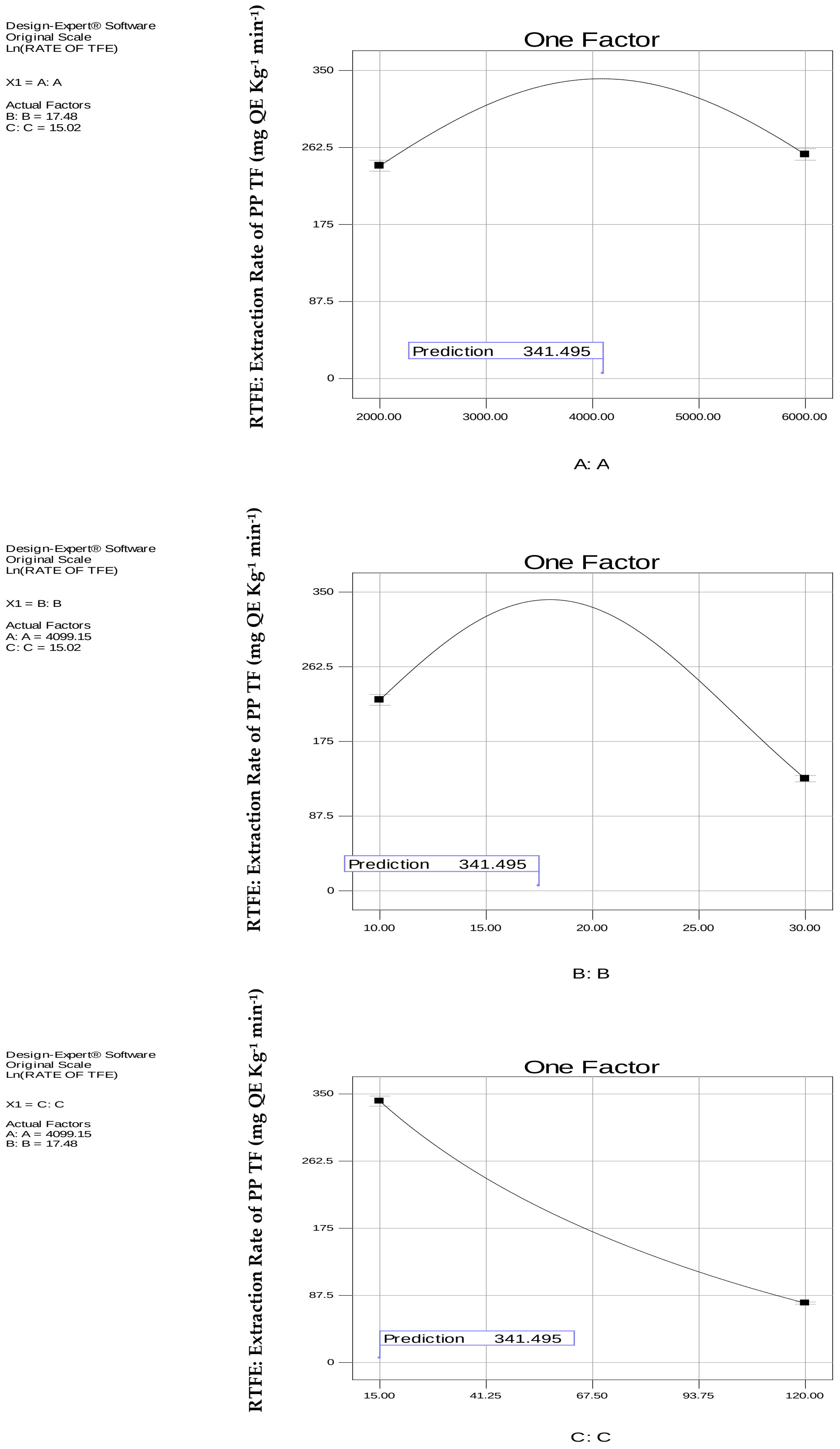

Figure S7. The effect of the individual extraction parameters A, B, C on the extraction rate of the PP total flavonoids.
